# Single‐EV Analyses Require Rigorous Antibody Qualification: PD‐L1 Profiling in Cell Models and Patient Plasma

**DOI:** 10.1002/jex2.70144

**Published:** 2026-05-25

**Authors:** Tobias Tertel, Fabiola Nardi Bauer, Oumaima Stambouli, Sabine Kasimir‐Bauer, Corinna Keup, Stephan Lang, Ali Sak, Martin Stuschke, Tienush Rassaf, Martin Schuler, Stefan Kasper‐Virchow, Bernd Giebel, Alexander Schramm

**Affiliations:** ^1^ Institute for Transfusion Medicine University Hospital Essen University of Duisburg‐Essen Essen Germany; ^2^ Department of Medical Oncology West German Cancer Center University Hospital Essen University of Duisburg‐Essen Essen Germany; ^3^ Department of Gynaecology and Obstetrics University Hospital Essen University of Duisburg‐Essen Germany; ^4^ Department of Oto‐Rhino‐Laryngology University Hospital Essen University of Duisburg‐Essen Germany; ^5^ Department of Radiotherapy University Hospital Essen University of Duisburg‐Essen Essen Germany; ^6^ Department of Cardiology and Vascular Medicine West German Heart‐ and Vascular Center University Hospital Essen University of Duisburg‐Essen Germany; ^7^ National Center for Tumor Diseases (NCT) West Essen Germany

**Keywords:** antibody validation, cancer plasma, extracellular vesicles, imaging flow cytometry, PD‐L1, single‐vesicle analysis

## Abstract

Imaging flow cytometry (IFCM) has emerged as a powerful method for high‐throughput phenotyping of small extracellular vesicles (sEVs) at single‐vesicle resolution. However, the reliability and sensitivity of IFCM critically depend on the performance of the antibodies used. In this study, we systematically compared several commercially available anti‐PD‐L1 antibodies for their ability to detect PD‐L1‐positive sEVs. Despite being derived from the same clone, antibodies from different manufacturers showed striking differences in labelling efficiency. These findings demonstrate that antibody validation for cellular targets does not ensure suitability for single‐EV analysis and underscore the need for rigorous, application‐specific qualification. To address this, we developed a robust IFCM workflow in full alignment with MIFlowCyt‐EV reporting standards. Using a qualified anti‐PD‐L1 antibody, we achieved reproducible detection of PD‐L1^+^ sEVs directly in unprocessed plasma samples from tumour patients. To assess the quantitative potential of single‐EV analysis, we compared this approach with bead‐based capture and performed dilution experiments. While bead‐based methods yielded a relatively narrow range of mean fluorescence intensities across samples, IFCM revealed distinct PD‐L1^+^ sEV levels, supporting its semi‐quantitative capability. Finally, we applied this workflow to plasma samples from patients with head and neck, lung, and breast cancer, as well as healthy donors. In subsets of all three cancer cohorts, elevated PD‐L1^+^ sEV levels were observed, suggesting potential clinical relevance. While in‐depth clinical correlations lie beyond the scope of this study, our findings establish a validated, standardized protocol for IFCM‐based single‐EV profiling and highlight the central role of antibody quality in ensuring analytical accuracy.

## 1 Introduction

Small extracellular vesicles (sEVs), including exosomes, are nano‐sized, membrane‐enclosed particles released by virtually all cell types and found in all body fluids. Typically ranging from 70 to 150 nm in diameter, they transport proteins, lipids, and nucleic acids that reflect their cellular origin (Yáñez‐Mó et al. 2015). sEVs have attracted considerable interest as potential biomarkers for various diseases, including cancer (Fais et al. 2016). In oncological contexts, they have been associated with tumour progression, metastasis, immune evasion, and therapy resistance, partly through the modulation of epithelial‐to‐mesenchymal transition, immunosuppressive signalling and angiogenesis (Kalluri and McAndrews 2023).

A well‐established axis of tumour immune evasion involves the interaction between the checkpoint receptor PD‐1 and its ligand PD‐L1. PD‐L1, often expressed by tumour cells, suppresses cytotoxic T cell activity and limits antitumour immunity. Antibodies targeting PD‐1 or PD‐L1 have thus become key components of modern immunotherapy in various malignancies (Franklin et al. 2017; Topalian et al. 2023). Beyond the tumour cell surface, PD‐L1 has also been detected on sEVs, where it may contribute to systemic immunosuppression or serve as a surrogate marker of tumour activity (Chen et al. 2018; Eslami‐S et al. 2024). These findings have motivated efforts to quantify PD‐L1^+^ sEVs in blood as potential indicators of immune checkpoint activation or tumour burden.

However, these translational efforts are hampered by substantial methodological challenges. Current single‐sEV technologies face notable limitations, as no standardized method yet exists for reliably quantifying and phenotyping sEVs at the single‐vesicle level. Techniques such as nanoparticle tracking analysis (NTA), resistive pulse sensing, or flow cytometry based on light scatter provide information on particle size and concentration but cannot reliably distinguish sEVs from other small, co‐isolated particles such as lipoproteins or protein aggregates (Dragovic et al. 2011; Droste et al. 2021; Giebel and Helmbrecht 2017; Sokolova et al. 2011). These limitations are due in part to the small size and low refractive index of sEVs, which result in weak light scattering and low detection sensitivity.

As a result, fluorescence‐based approaches have become increasingly important, as they allow for molecularly specific detection with fluorochrome dyes or fluorochrome‐conjugated antibodies (Tertel et al. 2022). When combined with high‐sensitivity platforms, such as fluorescence microscopy or advanced cytometry, these methods enable the detection of vesicles even below the diffraction limit of conventional optics. However, limited epitope density and surface area on individual vesicles can impair antibody binding, and unbound fluorochromes contribute to high background signals, necessitating extensive purification steps in conventional assays. This reduces throughput and limits reproducibility in complex biofluids.

To overcome these issues, imaging flow cytometry (IFCM) combines the statistical robustness of flow cytometry with the spatial resolution of fluorescence microscopy. It allows for direct, multiplexed analysis of single vesicles in minimally processed samples and has been shown to reliably detect EVs under physiological conditions (Görgens et al. 2019; Ricklefs et al. 2019). Our previous work has optimized IFCM protocols to minimize sample manipulation while improving signal‐to‐noise ratios and labelling reproducibility (Tertel et al. 2020, Tertel et al. 2020). Recently, we further expanded this platform to perform comprehensive high‐plex surface protein profiling of sEVs (Holcar et al. 2025). Nonetheless, the antibody selection for reliable single‐EV analysis remains a significant challenge.

Despite increasing adoption of IFCM in EV research, comprehensive protocols for antibody benchmarking and validation remain scarce, and comparative studies across vendors are largely missing.

In this study, we tackled the challenge of labelling and detecting sEVs at the single‐vesicle level with anti‐PD‐L1 antibodies. We first evaluated a panel of commercially available fluorochrome‐conjugated anti‐PD‐L1 antibodies using engineered extracellular vesicles derived from immortalized mesenchymal stromal cells (ciMSCs) stably expressing PD‐L1 fused to enhanced green fluorescent protein (eGFP). This dual‐labelling system enabled robust benchmarking of antibody performance in IFCM. Following antibody selection, we compared IFCM‐based detection of PD‐L1^+^ sEVs in clinical plasma samples with an orthogonal bead‐based capture assay. Finally, we applied the optimized IFCM workflow to quantify PD‐L1^+^ sEVs in plasma from patients with head and neck, non‐small cell lung and breast cancer.

## 2 Material and Methods

### 2.1 Patients and Informed Consent

Blood plasma from patients with malignant conditions, as described in the main text, was obtained at University Hospital Essen. Use of patient material for the purpose of this study was approved by the Ethics committee at University of Duisburg‐Essen (19‐8812‐BO for head and neck cancer samples, 12‐5265‐BO for breast cancer samples and 18‐8364‐BO for lung cancer samples). Patient samples were included solely based on availability for this project. No selection with regards to treatment or tumour stages and subtypes was performed. All research activities were permitted by a scientific advisory committee at the West German Biobank (WBE; WBE‐077/19).

### 2.2 Generation of PD‐L1‐eGFP Expressing ciMSCs

To generate human clonal immortalized mesenchymal stromal cells (ciMSCs) expressing PD‐L1 linked to eGFP, we utilized a lentiviral transduction approach. The lentiviral vector pCL6EGwo (Leurs et al. 2003), containing the PD‐L1‐eGFP expression cassette, was employed to introduce the encoded genes into ciMSCs. This process was facilitated by lentiviral particles produced as previously described (Ludwig et al. 2018), utilizing the helper plasmid pCD/NL‐BH (Mochizuki et al. 1998) and the codon‐optimized plasmid encoding the human foamy virus envelope, pcoPE01 (Müllers et al. 2011).

ciMSCs were seeded and transduced in 24‐well plates (Cellstar, Greiner Bio‐One, Frickenhausen, Germany) at a density of 15,000 cells per well. Twenty‐four hours following the addition of viral particles, cells were washed twice with phosphate‐buffered saline (PBS) and replenished with fresh medium. The ciMSCs were cultured for an additional 48 h or until they achieved confluency. Upon reaching the desired density, cells were transferred to T75 flasks for further expansion. Next, PD‐L1‐eGFP expressing ciMSCs were enriched by fluorescence‐activated cell sorting (FACS) on an Aria IIIu platform (BD Bioscience, Heidelberg, Germany).

### 2.3 Cell Culture

PD‐L1‐eGFP^+^ ciMSCs were cultivated in Dulbecco's Modified Eagles Medium (DMEM) (PAN‐Biotech, Aidenbach, Germany) supplemented with 10% human platelet lysate (hPL) as described previously (Labusek et al. 2023). FaDu cells were cultured in DMEM low medium (PAN‐Biotech) supplemented with 10% FBS (Biochrom) and 1 x Penicillin/Streptomycin (Thermo Fisher Scientific, Darmstadt, Germany). Typically, 3 × 10^4^ cells were seeded per 6‐well plate in 3 mL fresh media. Conditioned media were collected when expanding cells reached confluency. For the sEV analyses, conditioned media were first centrifuged for 5 min at 900 × *g* and then for 15 min at 2000 × *g*. Obtained supernatants were directly used for down‐stream processes.

### 2.4 EV Preparation

PD‐L1‐eGFP^+^ ciMSC‐EVs were prepared from conditioned media by polyethylene glycol 6000 precipitation followed by ultracentrifugation, exactly as described previously (Kordelas et al. 2014; Ludwig et al. 2018).

### 2.5 EV Labelling

To characterize sEVs from different sources, specific staining protocols were established. Prior to IFCM or bead analysis, all samples were filtered through 0.22 µm syringe filters (Sartorius) to reduce larger particles and debris. For FaDu cell‐derived sEVs, 190 µL of cell culture supernatant was directly incubated with 10 µL of antibody solution. For PD‐L1‐eGFP^+^ ciMSC‐EVs (5 µL input) and patient plasma samples, aliquots were incubated with 10 µL of the respective antibody solution. For plasma analysis, input volumes were initially titrated (0.25, 0.5, 1, 2.5 and 10 µL) to determine the linear detection range, with 10 µL established as the standard input volume for subsequent cohort analyses. Five different, commercially available anti‐PD‐L1 antibodies were tested: Three of these antibodies belonged to clone 29E.2A3, one conjugated with APC from EXBIO (29E.2A3‐APC) and two conjugated with PE from EXBIO (termed hereafter 29E.2A3‐PEa) or BioLegend (termed hereafter 29E.2A3‐PEb). Additionally, we selected two PE‐conjugated antibodies from clone MIH1, sourced from eBioscience (termed hereafter MIH1‐PEa) and BD Bioscience (termed hereafter MIH1‐PEb). To ensure comparability, all anti‐PD‐L1 antibodies were applied at a constant mass of 0.04 µg per reaction during EV labelling, a concentration established beforehand under non‐limiting conditions to provide a robust molecular excess (Tertel et al. 2020). In our dilution experiments, the variable was the sample input rather than the antibody amount; that is, sample titration was performed at a constant antibody input. For plasma, the 10 µL input was selected based on the observed approximately linear response range. Before analyses and without including any post‐staining procedure, all samples were stained for 2 h at room temperature and subsequently diluted with PBS to a final volume of 100 µL. More details about the antibodies are given in Tables S1 and S2.

### 2.6 Imaging Flow Cytometry (IFCM)

All samples were measured using the built‐in autosampler from U‐bottom 96‐well plates (Corning Falcon, cat. no. 353077) with 5 min acquisition time per well on the AMNIS ImageStreamX Mark II Flow Cytometer (AMNIS/Luminex, Seattle, WA, USA). Data acquisition was triggered on the fluorescence channel (Ch03 for PE, Ch12 for APC) to specifically record antibody‐labelled events. This thresholding strategy intentionally excludes non‐fluorescent particles (e.g., non‐labelled EVs, background noise) to maximize sensitivity for the PD‐L1^+^ subset. The ‘removed beads option’ was deactivated (Görgens et al. 2019; Tertel et al. 2020). Obtained data were analysed as described previously (Tertel et al. 2020). Detailed machine settings are provided in Table S3.

### 2.7 Bead Capturing

Aliquots of functional Bead C6 (BD Biosciences, Heidelberg, Germany) were loaded with unconjugated anti‐PD‐L1 antibody (BioLegend, clone 29E.2A3) according to the manufacturer's protocol. The antibody‐loaded beads were either incubated with 10 µL aliquots of the given plasma samples or 5 µL aliquots of obtained ciMSC‐EV preparations. After 2 h, the loaded beads were incubated with PE‐conjugated anti‐PD‐L1 antibodies (BioLegend, clone 29E.2A3) for 1 h. Before analysis on a Cytoflex S flow cytometer (Software CytExpert 2.3, Beckman‐Coulter), samples were washed twice with 1 mL of PBS.

### 2.8 Control Experiments for MIFlowCyt‐EV Standardization

To ensure compliance with MIFlowCyt‐EV standards, several controls were implemented (Welsh et al. 2020). For the detergent controls, NP40 was added for 30 min to a final concentration of 1% (w/v) to the labelling reactions of both the single‐EV and bead‐capturing analyses. For Isotype controls, instead of antibodies, the respective isotype control was used (Table S2). However, matched isotype controls in identical formats were not uniformly available across all tested vendors. Moreover, because the non‐specific binding behaviour of isotype controls can vary substantially in complex EV analyses, we relied primarily on antibody‐only and detergent lysis controls as the most robust indicators to assess true assay background. Additionally, antibody‐only and EV‐only samples were measured. The device was calibrated using MESF beads (Bangs Laboratories, Inc., PE Cat: 827A, and APC Cat: 823).

### 2.9 Statistical Analyses

The statistical analysis and graphical representations were performed using GraphPad Prism (version 10.4.1, GraphPad Software, Boston, MA). Median values and ranges are provided. Statistical significance between groups was determined using the Kruskal–Wallis test followed by Dunn's multiple comparisons test. A *p* value < 0.05 was considered statistically significant.

## 3 Results

### 3.1 Commercial Antibodies Vary in Their Efficiency in the Labelling of PD‐L1‐eGFP Containing EVs

As we anticipated that flow cytometric analysis of sEVs is highly dependent on the quality of the antibodies used, we evaluated five distinct anti‐PD‐L1 antibodies for detection of PD‐L1^+^ EVs. Among these antibodies, three were derived from clone 29E.2A3: one was conjugated with APC, designated as 29E.2A3‐APC while two antibodies were conjugated with PE from different manufacturers, referred to as 29E.2A3‐PEa and 29E.2A3‐PEb. Additionally, we tested PE‐conjugated antibodies from the MIH1 clone from two separate companies, termed MIH1‐PEa and MIH1‐PEb. To enable unbiased benchmarking, all antibodies were used at the same mass input per reaction.

**Figure 1:**
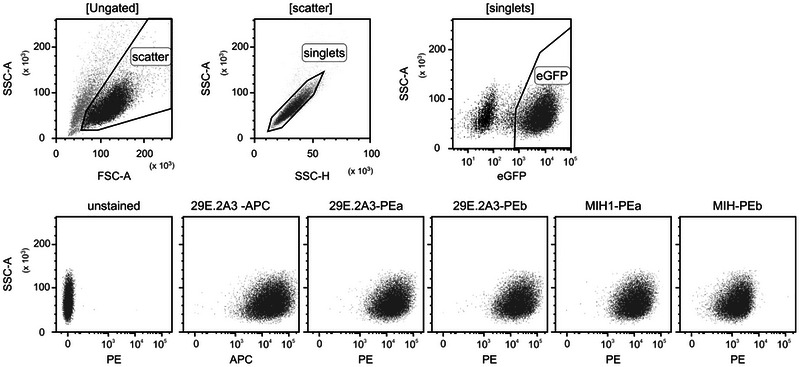
Different, commercially available anti‐PD‐L1 antibodies robustly detect PD‐L1 molecules on PD‐L1 expressing cells. Clonally expanded immortalized mesenchymal stroma cells (ciMSCs) genetically engineered to express PD‐L1‐eGFP served as a cell source for anti‐human PD‐L1 antibodies. After labelling with each of five different antibodies, cells were washed and analysed by flow cytometry. To focus on individual, living, PD‐L1‐eGFP expressing ciMSCs, cells were gated regarding their light scattering features (FSC‐A and SSC‐A), on singlets and on eGFP^+^ cells. The fluorescence intensity of the fluorochromes conjugated with the bound antibodies, PE or APC, is plotted against the intensity of the side scattered light (SSC‐A).

To confirm their specificity for PD‐L1 binding, we genetically engineered immortalized cells of a monoclonal mesenchymal stromal cell (ciMSC) line to express a PD‐L1‐eGFP fusion protein. These PD‐L1‐eGFP ciMSCs were labelled with each of the five antibodies and analysed by conventional flow cytometry. Among the tested antibodies, four demonstrated comparable contrast, that is, the fluorescent intensity of antibody‐labelled cells relative to unlabelled cells, while the contrast for the MIH1‐PEb antibody was noticeably weaker (Figure 1).

**Figure 2:**
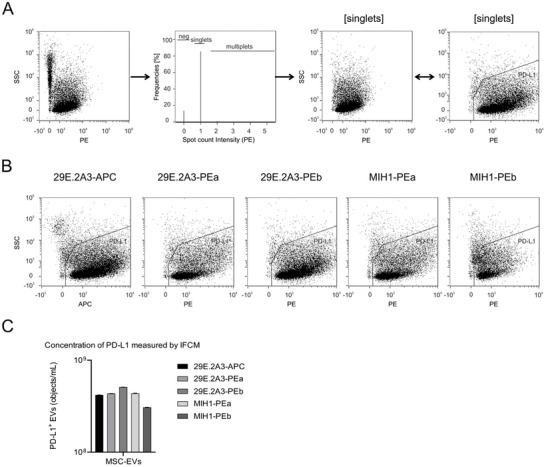
Commercial anti‐PD‐L1 antibodies vary in their EV labelling capabilities, even if they were manufactured from the same hybridoma cell clone and conjugated with the same fluorochrome, but manufactured by different companies. Equal amounts of EVs prepared from conditioned media of PD‐L1‐eGFP expressing ciMSCs were labelled with each of five different commercial anti‐PD‐L1 antibodies and analysed without any post labelling procedure by imaging flow cytometry. (A) Applied gating strategy shown exemplarily for PE‐labelled samples: of all recorded events, only those were considered for analyses for which the spot count feature detects exactly one object for any of the recorded events (2^nd^ plot; singlets). The PE intensity (PE) of ungated events and singlets were plotted against the size reflecting side scatter signal (SSC). The 4th plot is a zoomed‐in image of the 3rd plot and also contains the gate for the PD‐L1^+^ considered objects. (B) Comparison of recorded singlet objects following labelling with any of the given anti‐PD‐L1 antibodies. To enable semiquantitative comparison of the recorded object concentrations, samples were acquired for the same fixed time period. (C) The concentration of the recorded PD‐L1^+^ objects within each of the labelled EV samples is compared in the bar graph depicting the concentration as objects/mL. Statistical analysis for the antibody‐screening comparison in Panel C (Kruskal–Wallis test) did not reveal significant differences among the groups (*p* > 0.05).

Next, to explore the antibodies' capability to label PD‐L1^+^ sEVs, we prepared sEVs from PD‐L1‐eGFP ciMSCs using PEG‐precipitation followed by ultracentrifugation and labelled them with either of the five different anti‐PD‐L1 antibodies (Ludwig et al. 2018). The specificity of the antibody labelling and the capability of the IFCM platform to detect single vesicular events were rigorously validated using the PD‐L1‐eGFP reference system (Figure S2A–C). Furthermore, visual inspection of single events confirmed the co‐localization of PD‐L1 and eGFP signals on individual vesicles, validating the single‐particle resolution of the assay (Figure S2C). Without performing any post‐labelling steps, the samples and controls (see Figure S1) were analysed by IFCM. While virtually no objects were detected in any of the control samples, antibody‐labelled single objects were detected with all five antibodies (Figure 2A,B). Based on the absence of background objects and the detergent sensitivity of the labelled objects, we considered them *bona fide* sEVs.

Consistent with the results obtained from cells, four antibodies labelled PD‐L1‐eGFP ciMSC‐sEVs more brightly than the fifth. The highest amount of labelled sEVs was detected using the 29E.2A3‐PEb antibody, while three antibodies, 29E.2A3‐PEa, 29E.2A3‐APC and MIH1‐PEa resulted in comparable concentrations of labelled sEVs (Figure 2C). The concentration of labelled sEVs was notably lower in samples labelled with the MIH1‐PEb antibody, in accordance with the lower labelling intensities observed on cells. Thus, while all antibodies demonstrated anti‐PD‐L1 reactivity, their labelling properties varied markedly, even among PE‐labelled antibodies of the same clones from different manufacturers.

### 3.2 Only Selected Commercial Antibodies Label FaDu Cell‐Derived EVs Sufficiently for IFCM Analyses

Since PD‐L1 overexpression may lead to supraphysiological levels of PD‐L1 epitopes on the surface of corresponding sEVs, we next explored whether the antibodies were also capable of detecting PD‐L1 on native sEVs. To this end, we assessed their ability to label sEVs released by PD‐L1‐expressing FaDu cells, a hypopharyngeal carcinoma cell line (Rangan 1972). FaDu cell culture supernatants were individually labelled with the five different anti‐PD‐L1 antibodies and subjected to IFCM analysis without post‐labelling.

**Figure 3:**
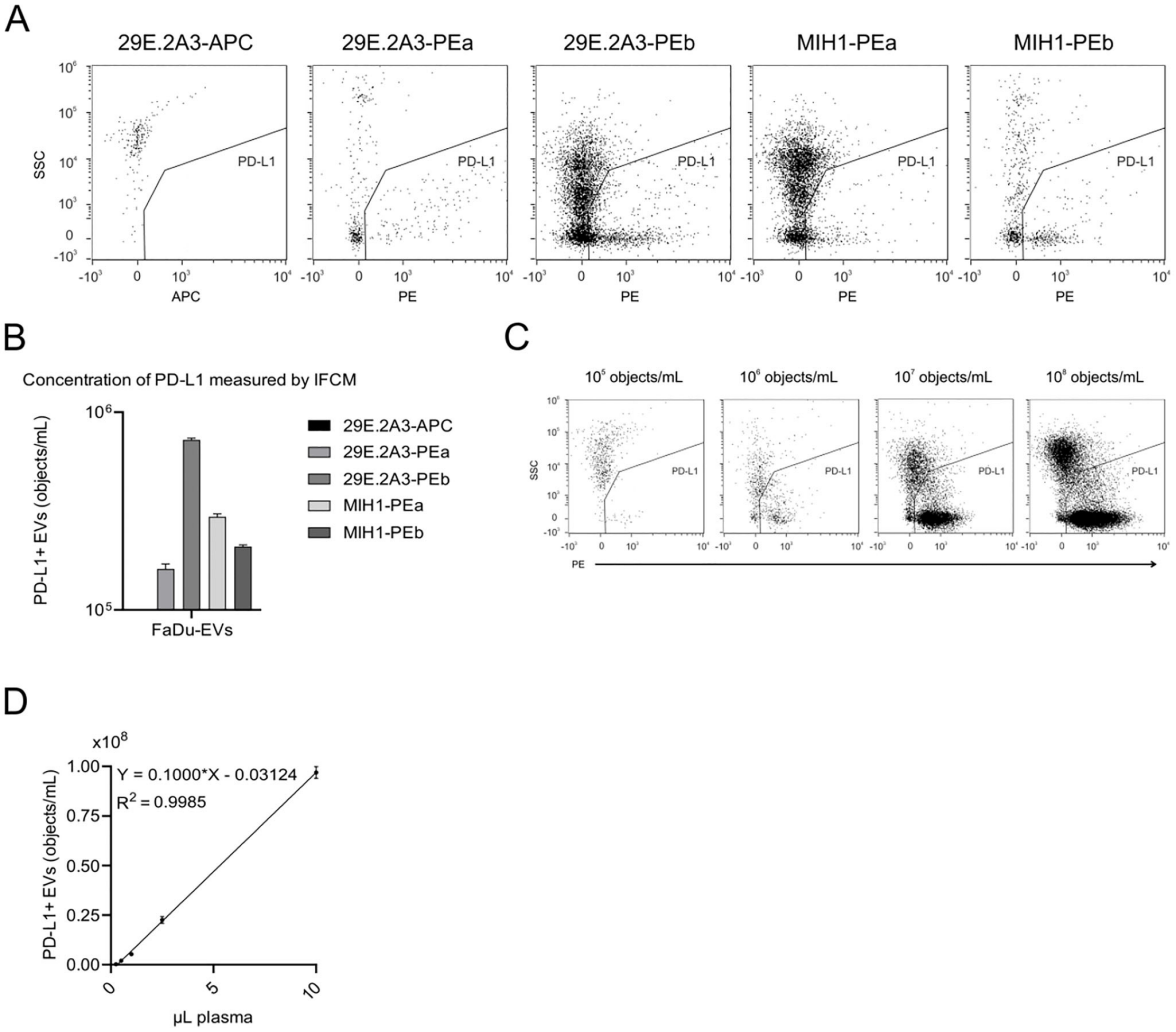
One of the five commercial antibodies enabled superior semiquantitative detection of PD‐L1^+^ sEVs derived from FaDu cells. EV preparations from FaDu cells were used to evaluate anti‐human PD‐L1 antibodies from different manufacturers and clones. (A) Comparative analysis of the performance of the tested anti‐PD‐L1 antibodies for detecting PD‐L1^+^ sEVs derived from FaDu cells. Data acquisition was performed for a fixed time period of 5 min per sample to allow for direct comparison of relative abundances; thus, varying event counts reflect differences in antibody labelling efficiency. (B) Concentrations of PD‐L1^+^ sEVs are expressed as objects/mL for each antibody tested. (C) IFCM‐based detection of PD‐L1^+^ sEVs using the 29E.2A3‐PEb antibody across a broad plasma dilution series (0.25, 1, 2.5 and 10 µL). (D) Quantification of the full titration series (0.25, 0.5, 1, 2.5 and 10 µL of plasma), including linear regression analysis, used to assess detection performance and dynamic range of the selected antibody. For the antibody‐screening comparison shown in Panel B, statistical analysis (Kruskal–Wallis test) did not reveal significant differences among the groups (*p* > 0.05).

Using the same gating strategy as before, PD‐L1^+^ sEVs were identified by three of the five tested antibodies. Specifically, PD‐L1^+^ sEVs were detected in samples labelled with 29E.2A3‐PEb, MIH1‐PEa and MIH1‐PEb. Once again, the highest EV concentration was observed with the 29E.2A3‐PEb anti‐PD‐L1 antibody (Figure 3A,B). These consistent results highlight that not all antibodies targeting abundant EV epitopes reliably label sEVs for single‐vesicle analysis, especially when using the IFCM platform. Based on these results, we selected the 29E.2A3‐PEb antibody for downstream analyses, recognizing its superior performance in a representative tumour‐derived EV sample and its utility for methodological validation. Notably, throughout these assay evaluations, antibody‐only controls showed a near‐complete absence of events. This supports the interpretation that fluorescent antibody aggregates are unlikely to represent a major source of false‐positive signals.

### 3.3 Imaging Flow Cytometry, but Not Bead Capturing, Enables Semi‐Quantitative Analyses of PD‐L1^+^ sEVs in Plasma Samples

To evaluate the dynamic range and sensitivity of IFCM‐based detection of PD‐L1^+^ sEVs in plasma, we performed a titration series using 0.25, 0.5, 1, 2.5 and 10 µL plasma from a head and neck cancer patient with high PD‐L1^+^ sEV levels (Figure 3C,D). All samples were labelled with the 29E.2A3‐PEb antibody and analysed without post‐labelling. The number of PD‐L1^+^ sEVs detected increased proportionally with the plasma input volume, demonstrating the scalability and linearity of the detection method (Figure 3D). Specifically, we observed (mean ± SD): 2.43 × 10^5^ ± 3.89 × 10^3^ objects for 0.25 µL, 1.95 × 10^6^ ± 4.10 × 10^5^ for 0.5 µL, 5.29 × 10^6^ ± 3.00 × 10^5^ for 1 µL, 2.26 × 10^7^ ± 1.69 × 10^6^ for 2.5 µL and 9.64 × 10^7^ ± 3.01 × 10^6^ for 10 µL plasma input. Based on these results, we selected 10 µL plasma as the standard input volume for subsequent analyses, as it provides high detection sensitivity while avoiding excessive sample consumption. The clear detection of PD‐L1^+^ sEVs even at 0.25 µL underscores the analytical sensitivity of the IFCM approach and confirms its suitability for semi‐quantitative measurements across a broad dynamic range.

**Figure 4:**
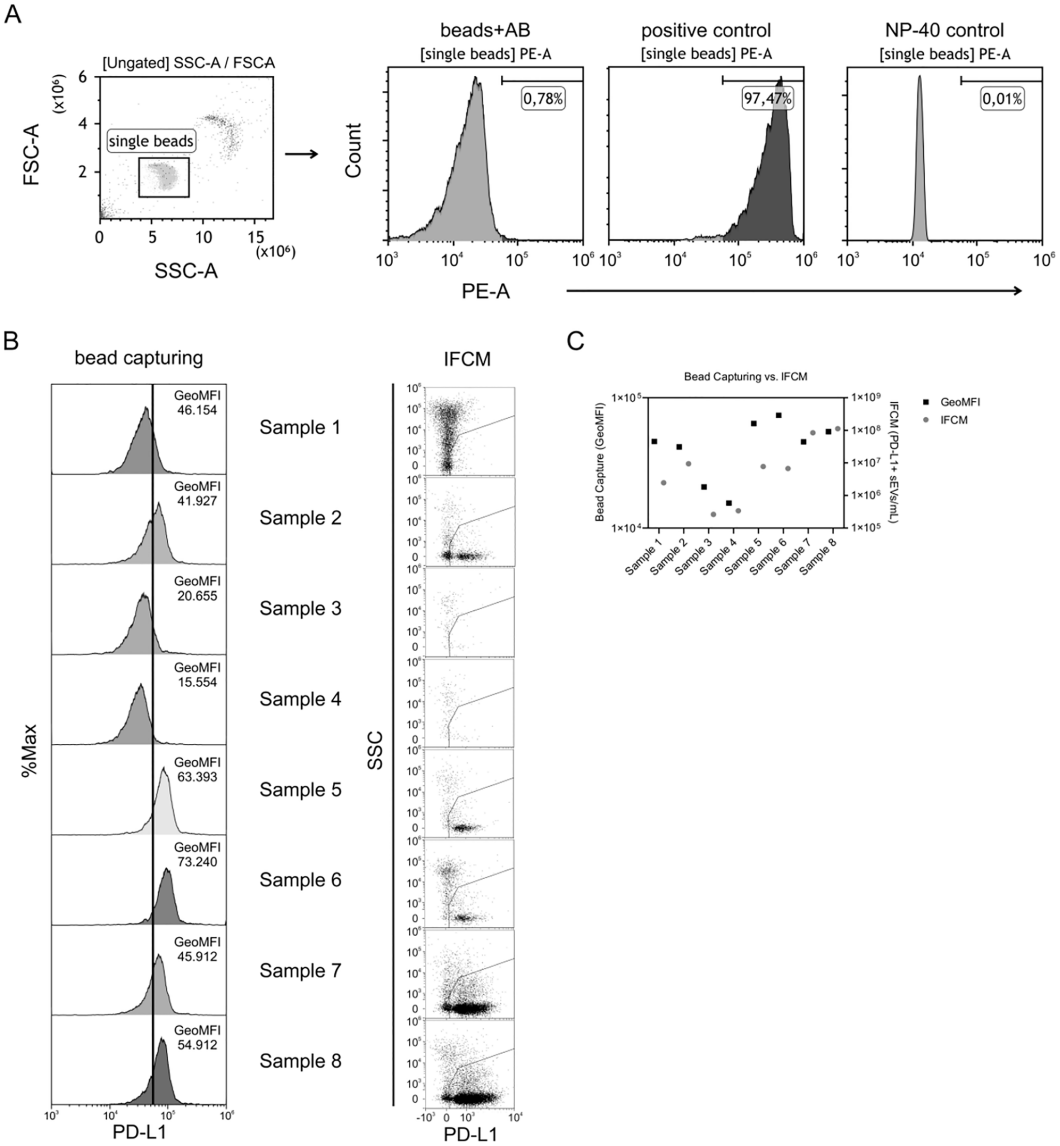
Comparison of Bead Capturing and Imaging Flow Cytometry (IFCM) for Detecting EV‐Bound PD‐L1. (A) Gating strategy for bead capturing, utilizing forward scatter (FSC) and side scatter (SSC) to identify single beads. The antibody‐only control is shown alongside a positive control (EVs derived from conditioned media of PD‐L1‐eGFP‐expressing ciMSCs) and a detergent control (NP‐40). (B) Representative data for eight unselected HNSCC plasma samples. The left column displays fluorescence intensity profiles (histograms) for PD‐L1 capture beads exposed to plasma and counterstained with the 29E.2A3‐PEb antibody, with corresponding GeoMFI values provided within the plots. The vertical line indicates the fluorescence threshold, which was established empirically using a blank control consisting of bare capture beads incubated only with the fluorescent detection antibody and no EV sample. The right column shows the corresponding IFCM analyses (scatter plots of SSC vs. PD‐L1 signal) for the same samples, illustrating the detection of distinct PD‐L1^+^ sEV populations. (C) Graph illustrating the direct comparison between the GeoMFI derived from bead capturing and the absolute concentration of PD‐L1^+^ sEVs determined by IFCM across all eight samples.

Next, we compared the IFCM methodology with bead‐capturing performance for analysing PD‐L1^+^ sEVs in patient plasma. To prepare suitable anti‐PD‐L1 antibody‐loaded beads, we conjugated beads with unlabelled anti‐PD‐L1 antibodies (clone 29E.2A3) and evaluated their effectiveness in capturing sEVs from the conditioned media of PD‐L1‐eGFP ciMSCs. After counterstaining with the 29E.2A3‐PEb antibody, the beads exhibited a 50‐fold increase in mean fluorescence intensity (Figure 4A). Addition of detergent eliminated the fluorescence signal, confirming that the antibody‐loaded beads specifically detect PD‐L1^+^ sEVs.

For direct comparisons, eight unselected plasma samples from patients with head and neck squamous cell carcinoma (HNSCC) were analysed by either IFCM or bead capturing. Both methods indicated PD‐L1‐positive material across the analysed samples, but bead capture showed only limited variation in GeoMFI compared with the much broader quantitative spread resolved by IFCM (Figure 4B,C). Specifically, bead capturing resulted in a relatively narrow range of mean fluorescence intensities across the samples (GeoMFI ranging from 1.6 × 10^4^ to 7.3 × 10^4^), whereas PD‐L1^+^ sEV levels detected by IFCM spanned several orders of magnitude (ranging from 6.1 × 10^4^ to 1.1 × 10^8^ objects/mL) (Figure 4C). These findings support that bead‐based capture provides qualitative confirmation of PD‐L1‐positive material, whereas IFCM offers substantially greater resolution to differentiate between high‐ and low‐abundance samples.

### 3.4 Subsets of Plasma Samples From Different Cancer Cohorts Show Elevated PD‐L1^+^ sEV Levels Compared to Healthy Donors

**Figure 5:**
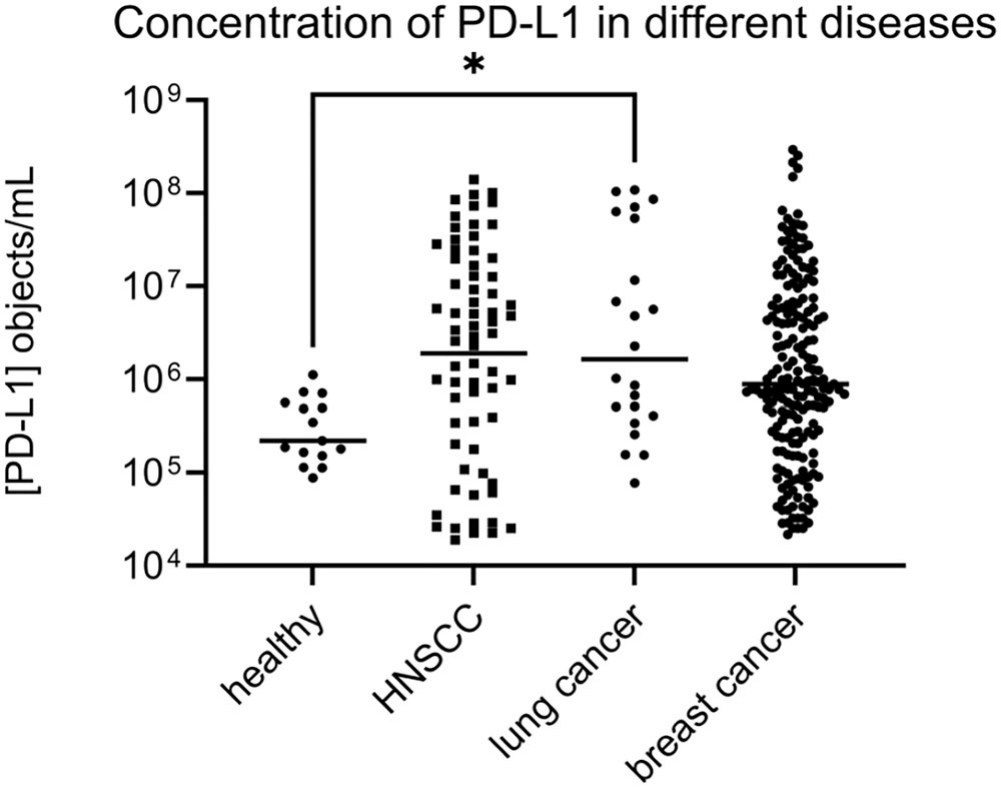
Plasma concentration of PD‐L1^+^ sEVs is elevated across distinct cancer types compared to healthy controls. Plasma samples from different cohorts were subjected to analysis using anti‐human PD‐L1 antibody 29E.2A3‐PEb. (A) The concentration of PD‐L1^+^ sEVs in plasma was analysed across healthy controls (*n* = 15), HNSCC (*n* = 74), lung cancer (*n* = 22) and breast cancer (*n* = 197). Statistical significance was determined using the Kruskal–Wallis test followed by Dunn's multiple comparisons test (*p < 0.05).

To assess platform robustness in primary blood samples and explore its suitability for biomarker discovery, we profiled PD‐L1^+^ sEV abundance across selected tumour entities with previously reported enrichment of PD‐L1^+^ EVs in patient plasma (Chen et al. 2018; Eslami‐S et al. 2024; Theodoraki et al. 2018). We used the validated IFCM‐based approach to evaluate PD‐L1^+^ EV levels in plasma samples from 74 HNSCC samples, 22 non‐small cell lung cancer (NSCLC) samples, and 197 breast cancer samples, and compared these to samples from 15 healthy donors. Consistent with published data, across all three tumour types investigated here, patients exhibited higher levels of PD‐L1^+^ sEVs compared to healthy donors. The median PD‐L1^+^ sEV concentration was 1.9 × 10^6^ EVs/mL (range 1.2 × 10^3^ EVs/mL – 1.4 × 10^8^ EVs/mL) in HNSCC patients, 1.6 × 10^6^ EVs/mL in NSCLC patients (range 7.8 × 10^4^ EVs/mL – 1.1 × 10^8^ EVs/mL), and 2.5 × 10^6^ EVs/mL in breast cancer patients (range 2.2 × 10^4^ EVs/mL – 3.0 × 10^8^ EVs/mL). In contrast, the median PD‐L1^+^ sEV concentration in healthy individuals was only 2.2 × 10^5^ EVs/mL (range 8.7 × 10^4^ EVs/mL – 1.1 × 10^6^ EVs/mL). This difference reached formal statistical significance for the NSCLC cohort (*p* < 0.05), while the HNSCC and breast cancer cohorts showed a strong trend (*p* = 0.075 and *p* = 0.071, respectively) towards elevated levels. Notably, 54% of HNSCC patients, 50% of NSCLC patients, and 45% of breast cancer patients had PD‐L1^+^ sEV concentrations > 1.1 × 10^6^ /mL (Figure 5A). In this dataset, PD‐L1+ sEV concentrations above 1.1 × 10^6^/mL were observed in tumour samples but not in healthy donor plasma. However, this study was not designed to establish PD‐L1^+^ sEV quantification as a biomarker. Validation of these findings would require prospective evaluation, which was beyond the scope of the present work.

## 4 Discussion

Reliable single‐vesicle analysis remains a key technical challenge in EV research, particularly when aiming to quantify immunologically relevant subsets such as PD‐L1^+^ sEVs. These EVs are increasingly recognized as potential biomarkers, providing insights into tumour immune evasion and therapy response (Chen et al. 2018; Eslami‐S et al. 2024; Serratì et al. 2022; Theodoraki et al. 2018). However, as our study highlights, meaningful data from such analyses critically depend on the rigorous validation of reagents, especially antibodies used for fluorescence‐based detection. While basic antibody reactivity can be initially confirmed at the cellular level, as demonstrated here using our engineered MSC models, bulk cellular validation does not reliably predict antibody performance in the nanoscale EV context. This fundamental biophysical disconnect justifies our strict focus on rigorous validation directly at the single‐EV level.

While the challenge of appropriate antibody selection is widely recognized in life sciences (Baker 2015; Bordeaux et al. 2010; Bradbury and Plückthun 2015; Nolan et al. 2022; Prince et al. 2021; Taussig et al. 2018; Voskuil 2017), it is surprising that anti‐PD‐L1 antibodies produced from hybridoma cells of the same clone by two different manufacturers, and conjugated with the same fluorochrome, displayed differing labelling efficacies for sEVs but not for cells. Although the basis of these differences was not investigated in detail, they may reflect variation in conjugation chemistry, degree of labelling, fluorochrome‐dependent effects on antigen binding, epitope accessibility, or formulation‐dependent properties of the antibody preparations. Together, these factors may contribute to the marked variation in EV labelling efficiency observed across commercially available reagents, even when antibodies are derived from the same clone (Nolan et al. 2022; Prince et al. 2021; Welsh et al. 2023; Welsh et al. 2020).

Beyond optimal antibody clone selection, several overarching biophysical and technical considerations must be addressed when establishing single‐EV workflows. First, the choice of fluorochrome is critical; brighter fluorophores, such as PE, are generally preferred over less bright options, such as APC, to maximize the detection of low‐abundance targets. Second, the spatial resolution of IFCM provides a unique advantage for rigorous quality control. By using the ‘Spot Count’ feature and exclusively gating on events with a single fluorescent spot (Spot Count = 1), we can effectively discriminate true single objects from coincidental swarms or background noise—a critical limitation in conventional, non‐IFCM. While traditional scatter‐based sizing via NTA is heavily confounded by matrix effects in unprocessed clinical samples, fluorescence‐triggered IFCM detection is driven primarily by accessible epitope number and fluorescence signal rather than physical size. While this approach does not provide orthogonal size characterization, and the detectable PD‐L1‐positive fraction may still be influenced indirectly by EV‐associated properties such as epitope abundance, it is less constrained by the physical size limitations of conventional scatter‐based detection.

Furthermore, while the single‐colour fluorescence triggering used in this study was motivated by the PD‐L1‐eGFP reference model, our standardized IFCM workflow is not restricted to single‐marker detection. In broader applications, modular multi‐colour panels can be implemented with acquisition settings configured to detect events across the relevant fluorescence channels, while limiting optical spillover and steric hindrance, as recently demonstrated in a comprehensive 25‐marker multiplex study (Holcar et al. 2025). Finally, potential steric hindrance must also be considered when comparing EV detection methods. In bead‐based capture assays, the macroscopic capture bead engages only a fraction of the nanoscale EV surface. While the opposing hemisphere may remain accessible for highly abundant targets, epitopes located at or near the bead‐EV contact interface become sterically masked. For low‐abundance targets such as PD‐L1, this spatial restriction is likely to limit the quantitative resolution of bead‐based assays. In contrast, our direct IFCM approach avoids solid‐phase capture, and thereby reduces interface‐related steric masking of PD‐L1 epitopes.

The qualified anti‐PD‐L1 antibody was further validated for single‐EV analysis by IFCM using clinically relevant plasma samples of tumour patients. To assess the quantitative capabilities of this approach, we established a titration series using different plasma input volumes. This revealed a linear increase in detected PD‐L1^+^ sEVs across a 40‐fold input range, with reliable detection already at 0.25 µL plasma. Based on these findings, we selected 10 µL as the standard input volume for subsequent analyses, balancing high sensitivity with reasonable sample consumption. These findings underscore the suitability of IFCM for robust and scalable semi‐quantitative EV phenotyping in clinical samples. Crucially, this single‐particle approach discriminates vesicular signals from soluble background.

To corroborate these findings, elevated levels of PD‐L1^+^ EVs within the same samples were orthogonally validated by immunocapture using anti‐PD‐L1‐coated beads, which were subsequently counterstained with fluorochrome‐conjugated anti‐PD‐L1 antibodies. In contrast to single‐vesicle analysis by IFCM, this bead‐based approach showed only a narrow range of GeoMFI variation, demonstrating limited quantitative resolution among the patient samples. A key limitation of bead‐based quantification lies in the fact that beads bind only a small fraction of available EVs before becoming saturated (Koliha et al. 2016; Koliha, Wiencek, et al. 2016). More quantitative applications may be achieved through careful dilution optimization or by using beads loaded with alternative capture antibodies. For instance, in a previous study, we employed anti‐CD63‐loaded beads to isolate PD‐L1^+^ sEVs (Jablonska et al. 2022). Due to the competitive binding of PD‐L1^+^CD63^+^ and PD‐L1^–^CD63^+^ sEVs, shifts in population ratios can affect labelling intensity when secondary fluorochrome‐conjugated anti‐PD‐L1 antibodies are applied. These bulk EV analyses are further complicated by differences in epitope abundance between vesicles, reinforcing the superior resolution and interpretability of IFCM‐based single‐EV detection.

To investigate PD‐L1^+^ sEV content in patient samples more comprehensively via IFCM, we applied this validated antibody to compare PD‐L1^+^ EV concentrations in plasma samples from healthy donors and patients across three disease cohorts. These cohorts, NSCLC, HNSCC, and breast cancer, consistently showed subsets of patients with elevated PD‐L1^+^ EV levels. Similar findings have been reported in other tumour entities, supporting the notion that increased PD‐L1 expression on circulating EVs is a recurrent phenomenon across cancers (Chen et al. 2018; Eslami‐S et al. 2024; Serratì et al. 2022; Theodoraki et al. 2018).

Our findings revealed that approximately half of the patients in each cohort exhibited clearly elevated levels of PD‐L1^+^ EVs, with concentrations reaching up to two orders of magnitude higher than the highest levels observed in healthy individuals. Similar patterns have been reported across independent studies: in melanoma, circulating exosomal PD‐L1 was higher in patients than in healthy donors and correlated with tumour burden and IFN‐γ activity (Chen et al. 2018). In HNSCC, bead‐captured plasma exosomes showed that PD‐L1 on EVs, but not soluble PD‐L1, tracked disease activity and stage (Theodoraki et al. 2018). In NSCLC, higher PD‐L1^+^ sEV concentrations associated with overall survival, and the combination with CTCs substantially strengthened risk stratification (Eslami‐S et al. 2024). Further melanoma data indicate that PD‐L1^+^ (and PD‐1^+^) EV populations can convey outcome‐relevant information when analysed as defined EV subsets (Serratì et al. 2022).

Collectively, these concordant observations support the view that quantitative PD‐L1^+^ sEV measurements capture clinically meaningful variation across cancers, all the more calling for rigorous selection of appropriate antibodies for single‐vesicle analysis.

## Conflicts of Interest

The authors declare no conflicts of interest.

## Supporting information




**Supporting Information**: jex270144‐sup‐0001‐SuppMat.docx

## Data Availability

The data that support the findings of this study are available from the corresponding author upon reasonable request.
