# Supplementary material for: Single‐EV Analyses Require Rigorous Antibody Qualification: PD‐L1 Profiling in Cell Models and Patient Plasma
Source: J Extracell Biol. 2026 May 25;5(5):e70144. doi: 10.1002/jex2.70144 (PMC13240509; doi:10.1002/jex2.70144)
Supplement: Supplementary file 1 — Supporting Information: jex270144‐sup‐0001‐SuppMat.docx [file JEX2-5-e70144-s001.docx]

**Supplementary Table 1: Antibodies used in this study.**

| antigens | conjugate | company | cat. | clone | Isotype |
| --- | --- | --- | --- | --- | --- |
| PD-L1 | APC | EXBIO | 1A-177-T100 | 29E.2A3 | Mouse IgG2b, κ |
| PD-L1 | PE | EXBIO | 1P-177-T100 | 29E.2A3 | Mouse IgG2b, κ |
| PD-L1 | PE | BioLegend | 329706 | 29E.2A3 | Mouse IgG2b, κ |
| PD-L1 | - | BioLegend | 329702 | 29E.2A3 | Mouse IgG2b, κ |
| PD-L1 | PE | eBioscience | 12-5983-42 | MIH1 | Mouse IgG1 |
| PD-L1 | PE | BD Bioscience | 557924 | MIH1 | Mouse IgG1 |

**Supplementary Table 2: Isotype controls.**

| Isotype | conjugate | company | cat. | clone |
| --- | --- | --- | --- | --- |
| Mouse IgG1 | PE | BD Biosciences | 555749 | MOPC-21 |
| Mouse IgG2b, κ | PE | Miltenyi Biotec | 130-120-698 | IS6-11E5.11 |
| Mouse IgG2b, κ | APC | Miltenyi Biotec | 130-122-932 | IS6-11E5.11 |

**Supplementary Table 3: Laser settings and detection channels/filters used.**

| Laser  [nm] | used Power  [mW] | max. Power  [mW] | Filter  [nm] |
| --- | --- | --- | --- |
| 375 | 70 | 70 | - |
| 488 | 100 | 100 | FITC (Ch02)  480-560 |
| 561 | 200 | 200 | PE (Ch03)  560-595 |
| 648 | 150 | 150 | APC (Ch11)  642-745 |
| 785 (SSC) | 70 | 70 | SSC (Ch06)  756-780 |


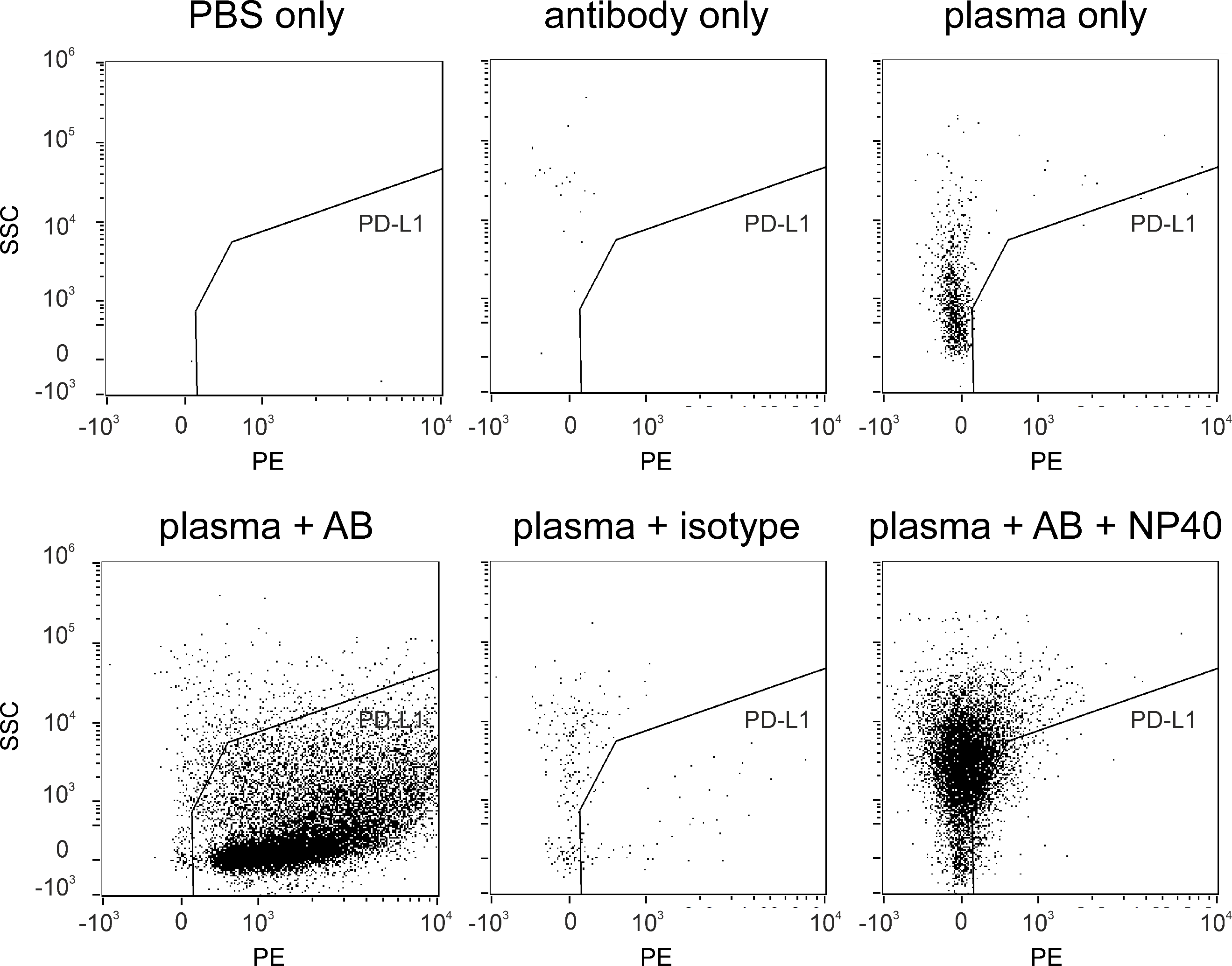


***Supplementary Figure 1. Representative dot plots for PD-L1⁺ sEV detection by imaging flow cytometry.*** *Representative density plots of control samples analyzed by imaging flow cytometry. Data acquisition was triggered on the fluorescence channel to exclude non-fluorescent particles. The plots display the side scatter (SSC) versus fluorescence intensity in the PE channel (Ch03), corresponding to the detection channel of the primary anti-PD-L1 antibody used in the main study (clone 29E.2A3-PEb).* ***(A)*** *PBS buffer control (Buffer only).* ***(B)*** *Antibody only control (no EVs).* ***(C)*** *Isotype control (Mouse IgG2b, κ-PE).* ***(D)*** *Detergent control (Sample + NP40). The absence of events in the relevant gates confirms that neither the buffer nor the antibody alone generates false-positive signals in the PE channel..*

*
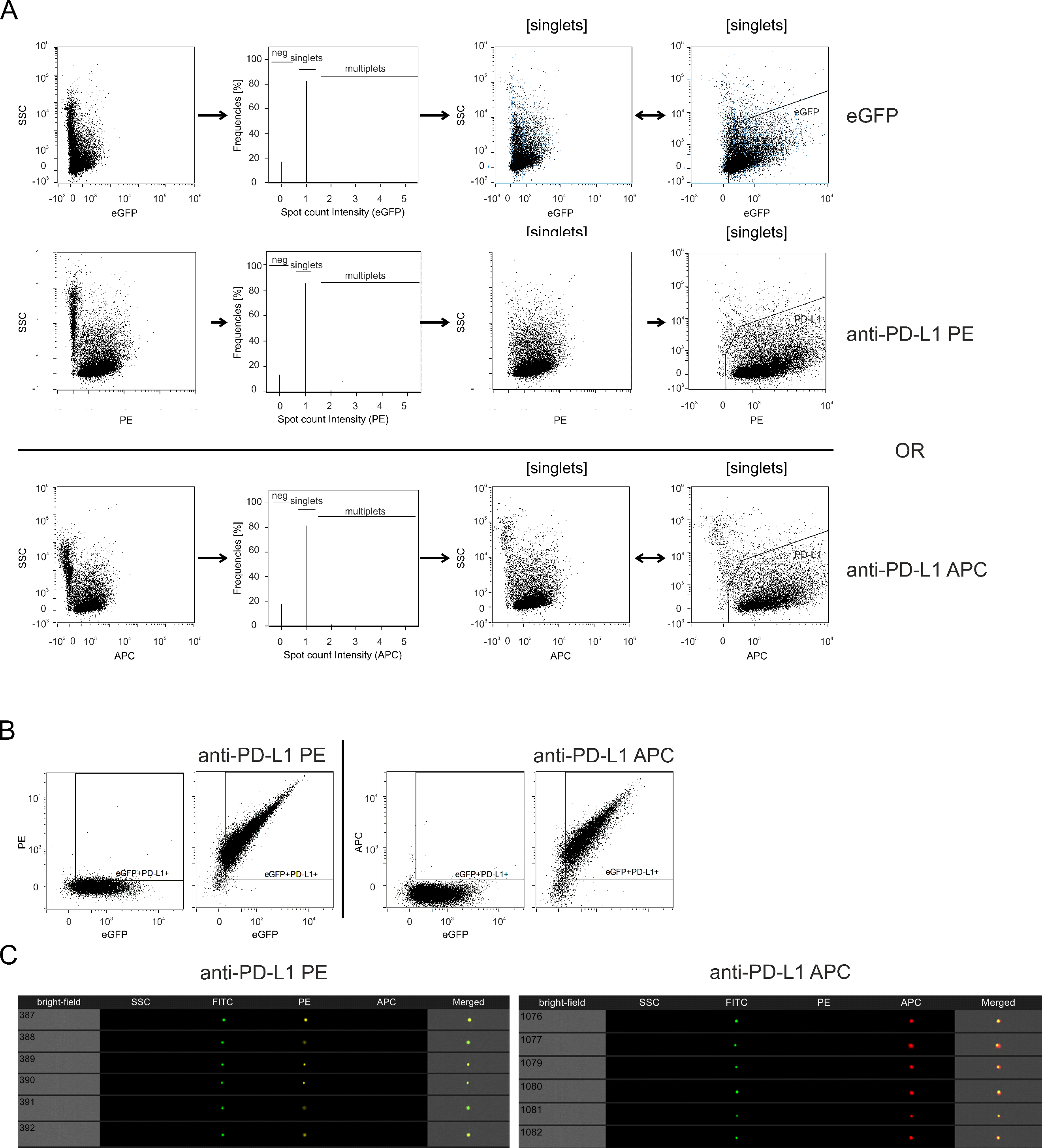
*

***Supplementary Figure 2: IFCM gating strategy and validation of antibody specificity using PD-L1-eGFP reference sEVs. (A)*** *Gating strategy for single sEV analysis. Objects were identified based on fluorescence thresholding in the respective detection channels (eGFP, PE, or APC). To ensure the analysis of single vesicles, a gate was applied to the "Spot Count" feature (Spot Count = 1), excluding background noise (Spot Count = 0) and coincidental swarms or aggregates (Spot Count > 1). The final plots display the identified single-vesicle populations utilized for downstream analysis.* ***(B)*** *Bivariate analysis of antibody specificity. sEVs derived from ciMSCs expressing a PD-L1-eGFP fusion protein were labelled with anti-PD-L1-PE (left) or anti-PD-L1-APC (right) antibodies. The diagonal double-positive populations (eGFP^+^/PD-L1^+^) demonstrate a direct correlation between the vesicular cargo (eGFP) and the surface marker (PD-L1), confirming specific antibody binding to vesicular objects.* ***(C)*** *Visual confirmation of single sEV detection. Representative image galleries of individual events from the double-positive populations shown in (B). Rows represent individual sEVs. Columns display channels for Brightfield, Side Scatter (SSC), eGFP (FITC), PD-L1-PE, PD-L1-APC, and the merged fluorescence image. The co-localization of eGFP and antibody signals on single spots visually confirms the detection of intact, individual extracellular vesicles.*
